# Prevalence of benign osseous lesions of the spine and association with spinal pain in the general population in whole body MRI

**DOI:** 10.1371/journal.pone.0219846

**Published:** 2019-09-09

**Authors:** Richard Kasch, Josephin Scheele, Mark Hancock, André Hofer, Christopher Maher, Robin Bülow, Jörn Lange, Andreas Lahm, Matthias Napp, Georgi Wassilew, Carsten Oliver Schmidt

**Affiliations:** 1 Center for Orthopedics, Trauma Surgery and Rehabilitation Medicine; Clinic and Outpatient Clinic for Orthopedics and Orthopedic Surgery, University Medicine Greifswald, Greifswald, Germany; 2 Faculty of Medicine and Health Sciences, Macquarie University, North Ryde, Sydney, Australia; 3 The University of Sydney, Sydney School of Public Health, NSW, Sydney, Australia; 4 Institute of Diagnostic Radiology and Neuroradiology, University Medicine Greifswald, Greifswald, Germany; 5 Center for Orthopedics, Trauma Surgery and Rehabilitation Medicine; Department of Trauma Surgery, University Medicine Greifswald, Greifswald, Germany; 6 Kliniken Maria Hilf Mönchengladbach, Academic Teaching Hospital of the RWTH Aachen, Mönchengladbach, Germany; 7 Institute for Community Medicine, Ernst-Moritz-Arndt University of Greifswald, Greifswald, Germany; Rush University Medical Center, UNITED STATES

## Abstract

**Background:**

Benign osseous lesions of the spine are common but precise population prevalence estimates are lacking. Our study aimed to provide the first population-based prevalence estimates and examine association with back and neck pain.

**Materials and methods:**

We used data from the population-based Study of Health in Pomerania (SHIP). Whole-body MRI examinations (1.5 Tesla: T1, T2, and TIRM weightings) were available from 3,259 participants. Readings of the spinal MRI images were conducted according to a standardized protocol by a single reader (JS). The intra-rater reliability was greater than Kappa values of 0.98. Pain measures included the seven-day prevalence of spine pain and neck pain, and average spine pain intensity due to spine pain during the past three months.

**Results:**

We found 1,200 (36.8%) participants with at least one osseous lesion (2,080 lesions in total). Osseous lesions were less common in men than in women (35.5% vs 38.9%; P = .06). The prevalence of osseous lesions was highest at L2 in both sexes. The prevalence of osseous lesions increased with age. Up to eight osseous lesions were observed in a single subject. Hemangioma (28%), and lipoma (13%) occurred most often. Sclerosis (1.7%), aneurysmal bone cysts (0.7%), and blastoma (0.3%) were rare. Different osseous lesions occurred more often in combination with each other. The association with back or neck pain was mostly negligible.

**Conclusion:**

Osseous lesions are common in the general population but of no clinical relevance for spinal pain. The prevalence of osseous lesions varied strongly across different regions of the spine and was also associated with age and gender. Our population-based data offer new insights and assist in judging the relevance of osseous lesions observed on MRIs of patients.

## Background

Benign osseous lesions of the spine are relatively common[1, 2] and include hemangiomas, lipomas, sclerosis, aneurysmal bone cysts, osteoid osteomas and osteoblastomas. While at the beginning of the last century autopsy was used to describe the prevalence and distribution of osseous lesions[1, 2] in the spine, it can now be performed non-invasively using T1 and T2 weighted magnetic resonance imaging (MRI)[3–6].

The most common type of benign osseous lesions are thought to be hemangiomas and lipomas[1, 2, 6]; however, precise population prevalence estimates are lacking due to the low number, quality and generalisability of existing studies. There are several key limitations to the existing literature. First, autopsy studies are not generalisable to the general population and do not provide good prevalence estimates across age groups. Second, no existing MRI study has included all regions of the spine from sampled subjects, making it difficult to accurately compare the prevalence across regions such as the lumbar or thoracic spine[1, 2, 6, 7]. Third, most previous studies have only reported on hemangiomas and lipomas so little is known about sclerosis, aneurysmal bone cysts, osteoid osteoma or osteoblastoma. Fourth, studies on the association with back pain have been limited to case reports,[8–19] and some retrospective studies[20–27] which are hard to generalize meaningfully.

Benign osseous lesions are usually observed as incidental findings derived from spine MR Imaging. They are commonly not regarded as a cause of pain but literature supporting this belief is lacking. Some osseous lesions may be associated with pain and some may result in fractures or neurological deficits. Therefore, there is a need to rigorously evaluate the association between different types of osseous lesions and spine pain[20, 28–30] in absence of acute symptoms or symptom progression. Their assessment in a general population sample avoids selection bias associated with a patient sample.

In conclusion, the prevalence, distribution, and clinical importance of benign osseous lesions in the spine remain unclear in the general population. Consequently, the aims of the current study were: 1) To describe the prevalence of osseous lesions, including hemangioma, lipoma, sclerosis, aneurysmal bone cysts, osteoid osteoma and osteoblastoma in a large general population sample; 2) To describe the distribution of osseous lesions across different regions of the entire spine and 3) To assess the association between osseous lesions and back or neck pain.

## Methods

### Design and Sample

**Fig 1:**
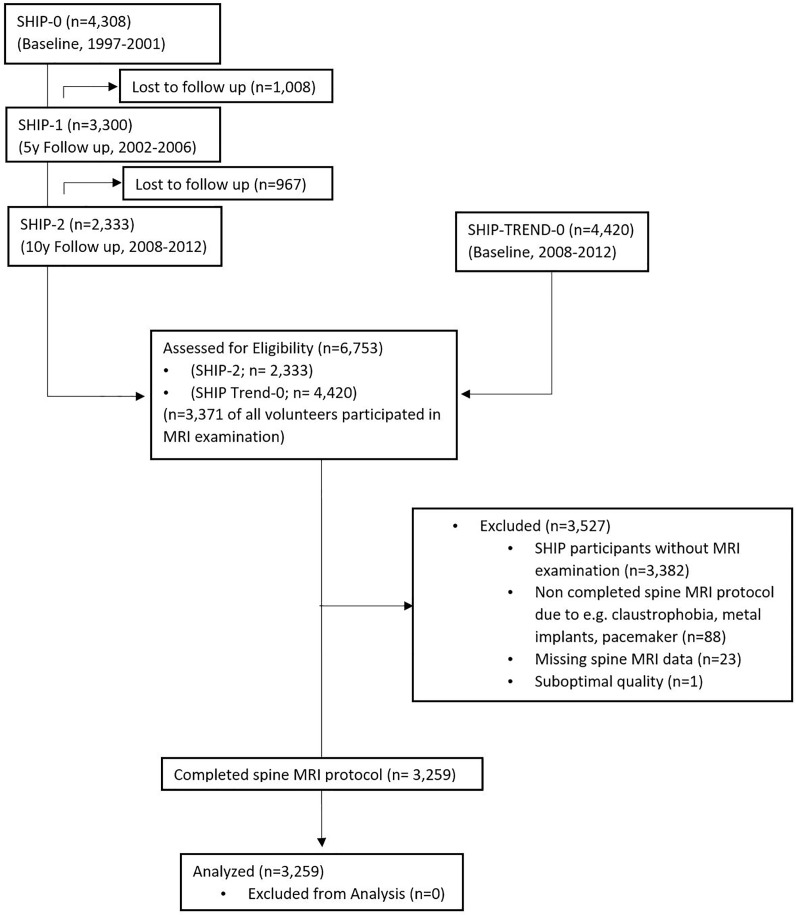
STROBE flow diagram: Flowchart of eligible participants for the current study.

We used, for this population-based evidence level 1 prognostic study, data from the Study of Health in Pomerania (SHIP) [31, 32]. SHIP is a population-based project consisting of two independent cohorts, SHIP and SHIP-TREND. Participants were recruited from the counties of North- and Eastern Pomerania including the two cities of Greifswald and Stralsund in North-East Germany (Fig 1).

Out of a net sample, excluding migrated or deceased persons, of 6,265 eligible individuals, 4,308 (2,192 women) participated (response 68.8%) in the baseline assessment of SHIP (SHIP-0). SHIP-0 examinations were performed from 1997–2001. The first and second follow-up examinations took place between 2002 and 2006 (SHIP-1, N = 3300) and 2008–2012 (SHIP-2, N = 2333) respectively. A second cohort (SHIP-Trend) was established in 2008. A stratified sample of 10,000 was drawn from the central population registry. Stratification variables were age, sex, and city/county of residence. Out of the net sample of 8,826, after exclusion of deceased and relocated subjects, 4,420 (2,275 women) participated (response 50.1%). The invitation procedure always comprised three written invitations, phone calls, and one personal contact. More details on the recruitment has been described elsewhere[32, 33].

We included all SHIP-2 and SHIP-Trend participants with a completed spinal protocol of the whole-body MRI. In total 3,371 out of 6,753 SHIP-2 and SHIP-Trend participants took part in the MRI examination out of which 3,259 completed the spinal MRI and were therefore eligible for the current study (Fig 1).

All participants provided written informed consent and the local Ethics Committee of the Medical University of Greifswald approved the study protocol (Reg.-Nr. BB 39/08a).

### MRI protocol

Spine imaging was performed as a part of a population-based whole body MRI study at 1.5-Tesla (Magnetom Avanto; Siemens Medical Solutions, Erlangen, Germany). Four trained technicians performed standardised examinations. The whole body MRI protocol was identical for all participants and included a plain whole-body MRI as well as detailed examinations of the head, neck, chest, abdomen, pelvis, and spine. The complete imaging protocols and extensive informed consent procedures have been described previously in detail[31, 34]. The present study analyzed native T1 and T2 weighted spine images in sagittal orientation as well as coronary turbo inversion recovery magnitude (TIRM) sequences of the whole body. The T1 and T2 weighted images were acquired with a voxel size of 1.1×1.1×4 mm at two stations of the spine. T1 weighted images were performed using the following imaging parameters: time of repetition (TR), 676 ms; time of echo (TE), 12 ms; flip angle (FA), 150°; matrix, 448x448; bandwidth, 151 Hz/pixel. T2 weighted images were acquired with TR of 3760 ms; TE of 106 ms; FA of 180°; matrix, 448x448; bandwidth, 189 Hz/pixel. TIRM fat suppressed imaging was performed using TR of 4891 ms; TE of 65 ms; FA of 180°; matrix, 240x320; bandwidth, 150 Hz/pixel with a voxel size of 2.1×1.6×5 mm following a standardized protocol at 5 stations of the whole body.

### Image analysis for osseous lesions

**Fig 2:**
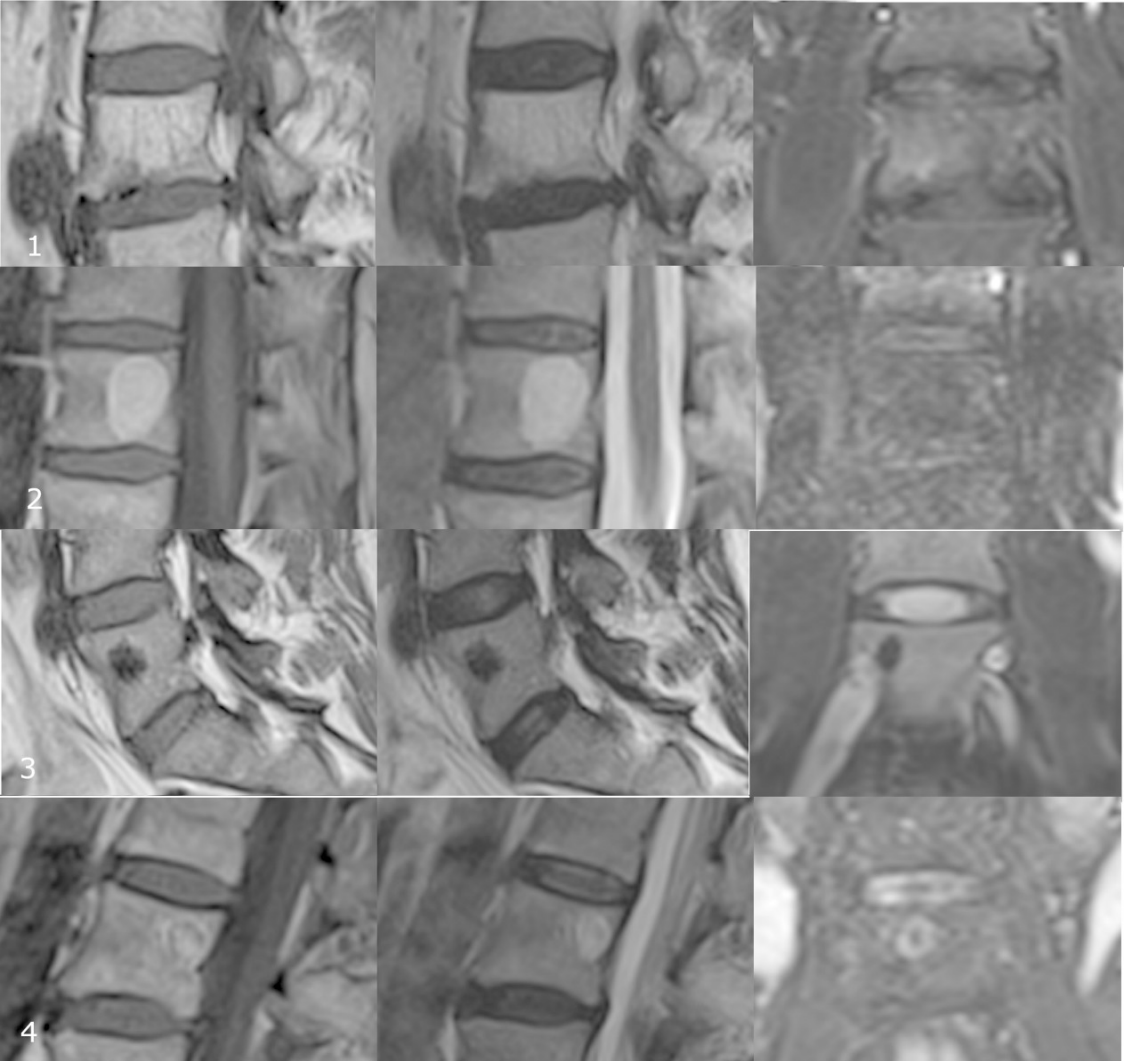
(1.) Examples for osseous lesions in MRI weighting of the vertrebra: 68Y old female with a hemangioma in a lumbar spine vertebrae 4. The osseus lesion is hyperintense in T1- and T2-weighted images and hyperintese as well as isointense in T2-weighted images with fat suppression and suggested as a fat-containing hemangioma with a typical striated pattern.; (2.) 74Y old female with a lipoma in thoracic spine vertebrae 12. The osseus lesion is oval, homogenous with hyperintense signal intensity in T1- and T2-weighted images and hypointense in T2-weighted images with fat suppression and suggested as an intraosseus lipoma.; (3.) 77Y old male with a sklerosis in thoracic spine vertebrae 5. The osseus lesion has a hypointense signal intensity in T1- and T2-weighted images as well as the T2-weighted images after fat suppression and suggested as a sklerotic osseus lesion or enostoma like in this case.; (4.) 68Y old female with a osteoblastoma in lumbar spine vertebrae 2. The osseus lesion has a low up to intermediate signal intensity on T1-weighted images with a low to predominantly high signal intensity in T2-weighted images without fat supression. In T2-weighted images with fat supression the osseus lesion reveals a hyperintense rim and a central hypointensity which could be interpreted as a nidus. This lesion might be suggested as an ostoidostoma and was categorized as an osteoblastom.

A single observer (JS) analyzed all spinal MRI examinations according to a standardized protocol blinded to all clinical characteristics of the participants. Findings were separately coded for each level of the spine (C1 to sacrum). In each of the three weightings, the signal intensity of osseous lesions was classified as hyperintense, hypointense or isointense to categorize the type of osseous lesion (Fig 2, and Table 1). Reading was performed using a digital picture archiving and communication system (IMPAX ES 5.2, AGFA Healthcare, Mortsel, Belgium).

**Table 1:** Matrix between signal intensity and osseous lesions in MRI weighting of the vertrebra.

	Hemangioma	Lipoma	Sclerosis	Aneurysmal bone cysts	Osteoblastoma
**Signal intensity in T1**	hyperintense	hyperintense	hypointense	hypointense	hypointense or isointense
	or isointense				
**Signal intensity in T2**	hyperintense	hyperintense	hypointense	hyperintense	hyperintense
	or isointense				
**Signal intensity in TIRM**	hyperintense	hypointens	hypointense	hyperintense and/or hypointense	hyperintense
	or isointense				

The test-retest reliability was assessed in a stratified-random sample of 200 SHIP participants more than one year after the initial reading. The stratification was designed to represent different types of benign osseous lesions (any osseous lesions, osseous lesions by spinal region, hemangioma, lipoma, sclerosis, aneurysmal cysts). End plate and bone marrow changes of the vertebral body like Modic types or lesions suspicious for malignancy were excluded as they were not part of this study. Kappa coefficients were computed for the presence of all types of osseous lesions. The intrarater reliability of MRI findings was greater than kappa of 0.98, indicating a high degree of agreement.

### Pain measures

Pain-related questions were administered as part of a standardized interview, comprising questions on spine pain and neck pain in the past seven days (entered into the analysis as yes/no). Furthermore, average spine pain intensity, and impairment of daily activities due to spine pain were assessed on a 0 to 10 scale using two items from a German version of the Chronic Pain Grade Scale (CPG)[35, 36] with a recall period of three months. We used the mean of the CPG pain intensity and impairment item as a measure of spine pain severity as recommended for population based samples[36]. Another item assessed sciatica as any radiating spine pain (entered into the analysis as yes/no).

### Statistical analysis

Standard descriptive statistics such as percentages and their confidence intervals were used to describe the prevalence of osseous lesions.

Poisson regression models were used to assess the association between binary pain outcomes (spine pain and neck pain in the past seven days, sciatica) and osseous lesions to estimate relative risks[37]. The association between spine pain severity and osseous lesions was computed based on two-part regression models[38] (using a probit regression model for the presence of spine pain, and a negative-binomial regression model for the severity of spine pain). The two-part model was used because of the zero inflated distribution of the outcome variable. Sex and age were entered in all regression models as covariates.

**Fig 3:**
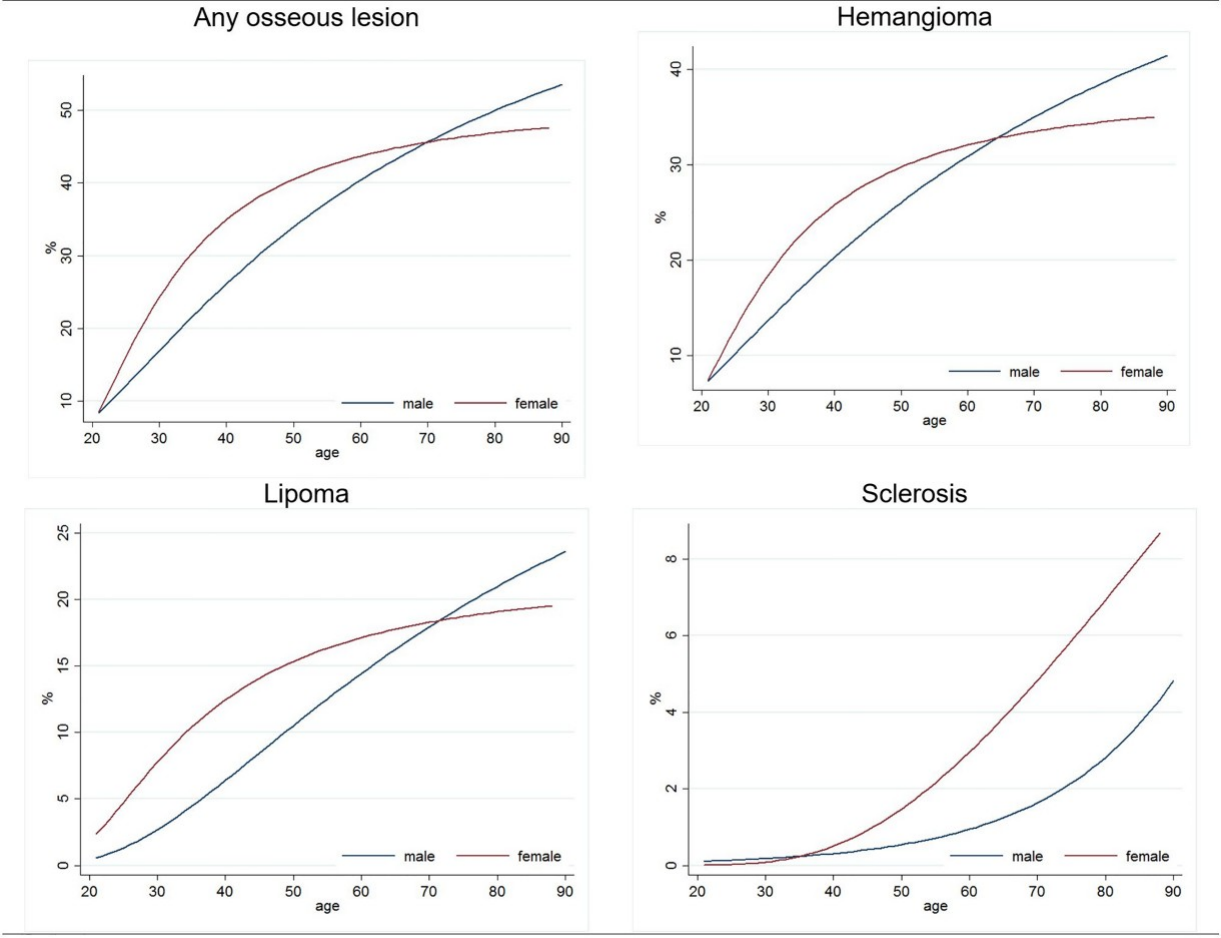
Age-specific prevalence rates of different types of benign osseous lesions; Based on logistic regression models. Non-linear age modelling was conducted with fractional polynomials (3 degrees of freedom, alpha = .1). Calculations were stratified by sex. Models were weighted with inverse probability weights to account for selective MRI participation. N = 3,259.

Inverse probability weights were used in all regression models as well as for the calculation of prevalence values to account for potential differential drop out related to the MRI examination. Probabilities underlying inverse probability weights have been derived from logistic regression models, using all variables from Table 1 as predictors. Statistical age-sex and age-spine pain interactions were entered in the model as well. Nonlinear associations were allowed for all continuous predictors using fractional polynomials[39] (with four degrees of freedom, and alpha set to 0.1 for the selection of powers). Age effects for Fig 3. were modelled using fractional polynomials as well (with three degrees of freedom, and alpha set to 0.1 for the selection of powers). Nonlinear age effects were not computed for aneurysmal bone cysts and osteoblastoma because of their low prevalence.

Because of weighting in this study Taylor-linearized variance estimation was applied. 95% CIs and p-values were estimated for two-sided tests. All analyses were conducted in Stata 13 (Stata Corp., College Station, TX).

## Results

### Sample characteristics

Sample characteristics of the 3,259 participants are described in Table 2. The mean age was 52.6 ±13.8 years. We found 1,200 participants with at least one benign osseous lesion (2,080 lesions in total). Overall, we identified 1,459 hemangioma, 512 lipoma, 77 sclerosis, 23 aneurysmal bone cyst, and 9 osteoblastoma. Almost one third of all participants experienced spine pain in the past seven days, or sciatica in the past three months, and one quarter reported neck pain in the past seven days.

**Table 2:** Sample characteristics.

	All N = 3,259	Men N = 1,623	Women N = 1,636
Age (years; SD)	52.6 (13.8)	52.9 (14.3)	52.2 (13.2)
Body mass index (mean; SD)	27.7 (4.4)	28.1 (3.7)	27.2 (5.0)
Educational level (%)			
- Less than 10 years	17%	18%	17%
- 10 years	55%	50%	59%
- More than 10 years	28%	32%	24%
Married (%)	81%	83%	78%
Back pain past 7 days (%)	30%	25%	35%
Neck pain past 7 days (%)	25%	18%	33%
Back pain severity past 3 months (mean; SD)	2.45 (2.49)	2.18 (2.39)	2.71 (2.56)
Sciatica past 3 months (%)	30%	26%	34%

SD: Standard deviation

### Prevalence and distribution of osseous lesions in the spine

The prevalence of benign osseous lesions stratified by sex varied substantially across different regions of the spine (Table 3). A total of 2.1% (N = 67) subjects had at least one lesion in the cervical vertebrae, 26.7% (N = 1,244) subjects had lesions in the thoracic vertebrae, 19.0% (N = 769) subjects had a lesion in the lumbar vertebrae, and 0.8% (N = 27) had a lesion in the sacrum. Osseous lesions were less common in men than in women (35.5% vs 38.9%, p = 0.06).

**Table 3:** Prevalence and distribution of osseous lesions stratified by sex.

	All N = 3,259	Men N = 1,623	Women N = 1,636	p-value*
	% (95% CI)	% (95% CI)	% (95% CI)	
**Distribution of osseous lesions**				
Cervical spine	2.1 (1.6 ; 2.8)	1.9 (1.3 ; 2.7)	2.4 (1.6 ; 3.5)	0.37
Thoracic spine	26.7 (25.1 ; 28.3)	25.5 (23.3 ; 27.7)	27.8 (25.6 ; 30.2)	0.14
Lumbar spine	19.0 (17.6 ; 20.5)	17.8 (16.0 ; 19.9)	20.0 (18.0 ; 22.2)	0.14
Os Sacrum	0.8 (0.5 ; 1.1)	0.9 (0.2 ; 1.4)	0.7 (0.4 ; 1.2)	0.56
**Type of osseous lesions**				
Hemangioma	28.1 (26.5 ; 29.7)	27.5 (25.3 ; 29.8)	28.7 (26.4 ; 31.0)	0.47
Lipoma	13.4 (12.2 ; 14.7)	12.1 (10.5 ; 13.8)	14.7 (12.9 ; 16.7)	0.04
Sclerosis	1.7 (1.3 ; 2.3)	1.0 (0.6 ; 1.6)	2.4 (1.7 ; 3.5)	<0.01
Aneurysmal bone cyst	0.7 (0.5 ; 1.2)	1.0 (0.3 ; 1.7)	0.5 (0.3 ; 1.1)	0.18
Osteoblastoma	0.3 (0.1 ; 0.5)	0.3 (0.1 ; 0.9)	0.2 (0.1 ; 0.6)	0.69
**Osseous lesions overall**	**37.3 (35.6 ; 39.0)**	**35.5 (33.2 ; 38.0)**	**38.9 (36.4 ; 41.4)**	**0.06**

*Calculated for sex differences based on Chi^2^ tests.

CI: Confidence interval

A single osseous lesion occurred in 20.9% of the sample, two lesions in 9.6% and more than two lesions in 6.7% (Table 4). At most, eight osseous lesions were observed in a single subject (Table 5).

**Table 4:** Prevalence of the number of osseous lesions and distribution in the spine.

Number of osseous lesions	Whole spine	Cervical spine	Thoracic spine	Lumbar spine	Os Sacrum
	% (95% CI)	% (95% CI)	% (95% CI)	% (95% CI)	% (95% CI)
0	62.7 (60.9 ; 64.4)	97.8 (97.2 ; 98.3)	73.3 (71.7 ; 74.8)	81.0 (79.5 ; 82.4)	99.2 (98.8 ; 99.4)
1	20.9 (19.5 ; 22.4)	2.1 (1.6–2.7)	18.4 (17.0 ; 19.8)	14.9 (13.6 ; 16.2)	0.8 (0.6 ; 1.2)
2	9.6 (8.5 ; 10.7)	<0.1 (0.0–0.2)	5.6 (4.9 ; 6.5)	3.2 (2.5 ; 3.9)	
3	3.8 (3.2 ; 4.6)		1.7 (1.3 ; 2.3)	0.8 (0.5 ; 1.3)	
4	1.4 (1.0 ; 1.8)		0.7 (0.5 ; 1.2)	0.1 (0.1 ; 0.3)	
5	1.0 (0.7 ; 1.5)		0.1 (0.1 ; 0.3)	0.1 (0.0 ; 0.2)	
6	0.3 (0.2 ; 0.6)		0.1 (0.0 ; 0.2)		
7	0.2 (0.1 ; 0.7)				
8	<0.1 (0.0 ; 0.2)				

CI: confidence interval; Empty cells: No observations.

N = 3,259

**Table 5:** Number of osseous lesions stratified by the type.

	Hemangioma	Lipoma	Sclerosis	Aneurysmal bone cyst	Osteoblastoma
Number of osseous lesions	% (95% CI)	% (95% CI)	% (95% CI)	% (95% CI)	% (95% CI)
0	71.9 (70.2 ; 73.5)	86.6 (85.2 ; 87.8)	98.3 (97.7 ; 98.7)	99.2 (98.8 ; 99.5)	99.7 (99.4 ; 99.9)
1	18.1 (16.8 ; 19.6)	11.0 (10.0 ; 12.3)	1.2 (0.8 ; 1.6)	0.8 (0.5 ; 1.2)	0.2 (0.1 ; 0.5)
2	5.8 (5.0 ; 6.7)	1.8 (1.4 ; 2.4)	0.3 (0.2 ; 0.6)		<0.1 (0.0 ; 0.2)
3	2.5 (2.0 ; 3.2)	0.4 (0.2 ; 0.7)	0.2 (0.1 ; 0.4)		
4	0.6 (0.4 ; 0.9)	0.1 (0.0 ; 0.2)	<0.1 (0.0 ; 0.2)		
5	0.6 (0.4 ; 1.0)	0.1 (0.1 ; 0.2)			
6	0.3 (0.1 ; 0.7)				
7	0.1 (0.0 ; 0.2)				
8	<0.1 (0.0 ; 0.2)				

Proportions, CI: confidence interval, Empty cells: No observations.

N = 3,259

**Fig 4:**
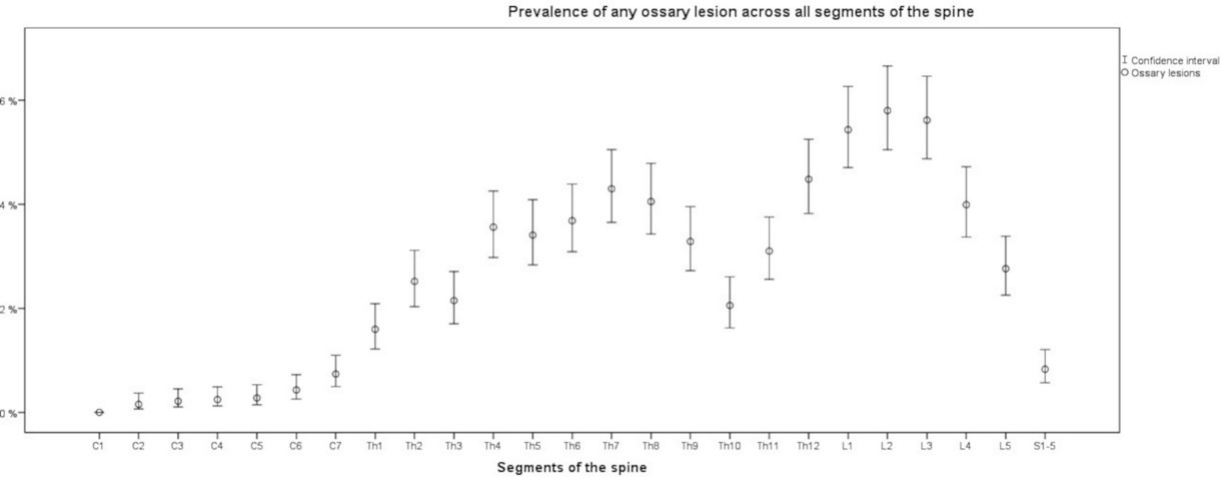
Prevalence of all benign osseous lesions on the segmental spinal scale; C1 –C7 = Benign osseous lesions in the cervical spine; Th1 –Th12 = Benign osseous lesions in the thoracic spine; L1 –L5 = Benign osseous lesions in the lumbar spine; S1-5 = Benign osseous lesions in the Os sacrum; Prevalence (95% CI); N = 3,259.

**Fig 5:**
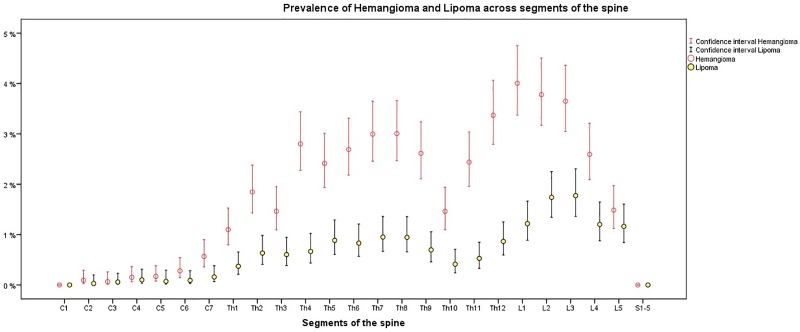
Prevalence of hemangioma and lipoma, including their lower and upper confidence intervals stratified on the segmental spinal scale; C1 –C7 = Osseous lesions in the cervical spine; Th1 –Th12 = Osseous lesions in the thoracic spine; L1 –L5 = Osseous lesions in the lumbar spine; S1-5 = Osseous lesions in the Os sacrum; Prevalence (95% CI). N = 3,259.

**Fig 6:**
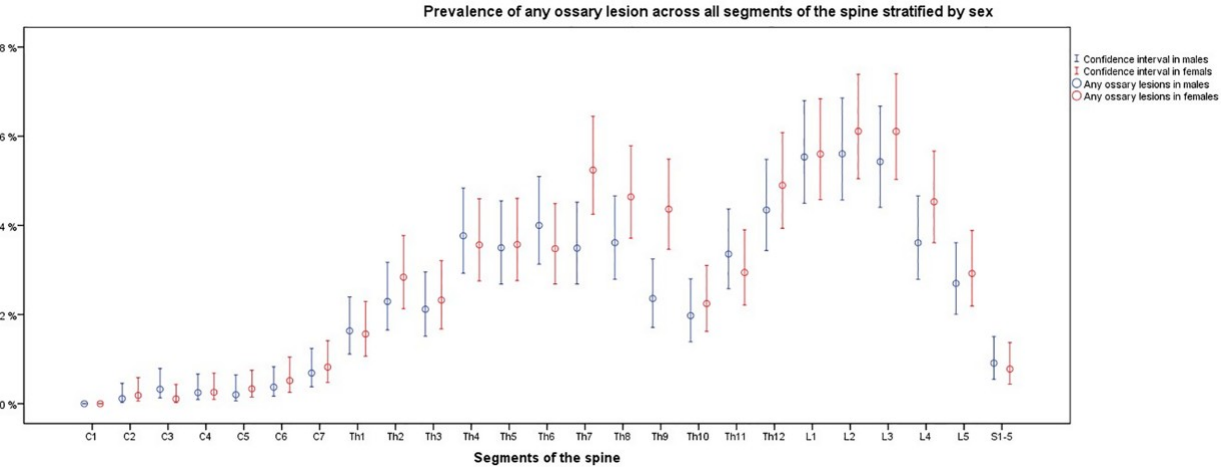
Prevalence of all osseous lesions, in both sexes and there lower and upper confidence intervals stratified on the segmental spinal scale; C1 –C7 = Osseous lesions in the cervical spine; Th1 –Th12 = Osseous lesions in the thoracic spine; L1 –L5 = Osseous lesions in the lumbar spine; S1-5 = Osseous lesions in the Os sacrum; Prevalence (95% CI). N = 3,259.

The prevalence of osseous lesions varied substantially across the segmental levels of the spine (Fig 4, Fig 5 and Fig 6). Prevalence was low in the cervical vertebrae with the highest prevalence occurring at C7. Prevalence was considerably higher in the thoracic and lumbar spine with peak prevalence at L2. Sacral prevalence was comparable with cervical findings and much lower than thoracic and lumber levels.

### Prevalence of different types of osseous lesions in the spine

The prevalence of hemangioma was highest (28.1%), followed by lipoma (13.4%). In contrast the prevalence of sclerosis (1.7%), aneurysmal bone cysts (0.7%), and osteoblastoma (0.3%) was low (Table 3). With the exception of aneurysmal bone cysts and osteoblastoma, females were affected more often than men. The largest relative sex effect concerned sclerosis (relative risk: 2.45 (95% CI: 1.34–4.45)), which was substantially more common in females. The prevalence of the different types of osseous lesions increased substantially with higher age (Fig 4). In males, we observed an almost linear increase for hemangioma and lipoma. In contrast a much steeper increase occurred in females in early adulthood (20–40), which levelled off in later years. In the eldest participants benign osseous lesions typically occurred more often in males compared to females. At all ages, sclerosis occurred more often in females.

Different benign osseous lesions occurred more often combined including lipoma in subjects with hemangioma (RR = 1.9, 95% CI: 1.5–2.3), sclerosis in subjects with hemangioma (RR = 3.3, 95% CI: 1.9–5.6) or lipoma (RR = 2.5, 95% CI: 1.4–4.6), and aneurysmal cysts in subjects with sclerosis (RR = 5.9, 95% CI: 1.4–25.0).

### Association of osseous lesions with pain-related measures

We evaluated the association between 10 types of osseous lesions and 4 measures of pain; and found only 2 of the 40 associations to be statistically significant (Table 6). The largest associations occurred between osseous lesions in the cervical spine and neck pain (relative risk: 1.55, 95% CI: 1.11–2.18), and between sclerosis and pain (relative risk: 1.44, 95% CI: 1.03–2.01). Of the remainder the associations were mostly negligible in magnitude and inconsistent regarding their direction.

**Table 6:** Association between osseous lesions with history of pain.

	Neck pain past 7 days	Spine pain past 7 days	Sciatica	Spine pain severity
	RR (95% CI)	RR (95% CI)	RR (95% CI)	ME (95% CI)
**Distribution of osseous lesions**				
Osseous lesions in the cervical spine	1.55* (1.11–2.18)	1.24 (0.87–1.76)	1.06 (0.73–1.54)	0.36 (-0.64–1.36)
Osseous lesions in the thoracic spine	0.88 (0.76–1.02)	0.94 (0.83–1.07)	0.97 (0.85–1.10)	-0.16 (-0.34–0.02)
Osseous lesions in the lumbar spine	0.98 (0.84–1.15)	1.01 (0.88–1.17)	1.10 (0.96–1.26)	0.06 (-0.16–0.28)
Osseous lesions in the Os sacrum	0.81 (0.38–1.72)	0.88 (0.47–1.65)	0.82 (0.42–1.60)	-0.32 (-1.19–0.55)
**Type of osseous lesions**				
Hemangioma	0.93 (0.80–1.07)	0.96 (0.85–1.09)	0.98 (0.90–1.10)	-0.08 (-0.27–0.10)
Lipoma	0.88 (0.72–1.07)	0.94 (0.79–1.12)	1.04 (0.89–1.22)	-0.02 (-0.27–0.22)
Sclerosis	1.45 (0.96–2.19)	1.44* (1.03–2.01)	1.27 (0.92–1.74)	0.38 (-0.69–1.46)
Aneurysmal bone cyst	0.86 (0.35–2.10)	0.96 (0.49–1.89)	1.30 (0.83–2.03)	-0.34 (-1.20–0.53)
Osteoblastoma	0.49 (0.07–3.35)	0.39 (0.06–2.55)	1.29 (0.56–2.96)	0.16 (-1.30–1.63)
**Osseous lesions overall**	**0.91 (0.80–1.03)**	**0.96 (0.85–1.07)**	**1.01 (0.90–1.13)**	**-0.06 (-0.23–0.11)**

Two part models were calculated for pain severity in the past 3 months, poisson models to derive relative risks (RR) for the other variables.

All models were adjusted for sex and age, and weighted for selective response to MRI participation.

ME: Average marginal effects for the predictor in the left column, in the scaling of the outcome variable.

* p<0.05, N = 3,259

## Discussion

### Main findings

The current study investigated the prevalence of benign osseous lesions in the general population, their distribution across different regions of the spine and associations with back and neck pain. Our results in a large general population sample offer new insights with regards to these aims. Benign osseous lesions are very common in the adult population. More than one third was affected; hemangioma and lipoma were the most common subtypes of osseous lesions. Among 44% of those affected, we counted more than one osseous lesion. Most osseous lesions occurred in the thoracic and lumbar spine. Considering the prevalence at each segmental level, the highest prevalence was found between L1 and L3, followed by a second but lower peak between T4 and T8. Furthermore, we observed a notable drop in prevalence at the thoraco-lumbar junction with the lowest prevalence at T10 in the thoraco-lumbar spine.

The prevalence of benign osseous lesions increased substantially with age. The graphical representation of age effects across sex strata indicates a higher prevalence of hemangioma and lipoma in younger females but the opposite in elder males. The age dependency for sclerosis differed in comparison to the other lesions as both sexes rises exponentially. We expect this to be a more degenerative finding unlike the others. The only substantial sex effect concerns sclerosis, which occurred more than twice as often in females compared to males.

Associations with back and neck pain were inconsistent, negligible in size and only 2 of the 40 associations examined were statistically significant. Contrary to earlier case reports and some retrospective studies, this indicates a rather low clinical importance of osseous lesions with regards to back and neck pain problems. The association of sclerosis and spine pain is consistent with the association between disc degeneration and low spine pain, and also age and disc degeneration previously reported [40, 41].

### Importance of results

Non-invasive T1 and T2 weighted magnetic resonance imaging (MRI) provides an excellent approach to estimate the prevalence of lesions in the spine. Our study was the first to implement this method in a large general population cohort to provide reliable prevalence estimates for osseous lesions. In concordance with previous studies we confirm that benign osseous lesions of the spine are relatively common and that the most common types of benign osseous lesions are hemangiomas followed by lipomas[1, 2, 6]. In line with earlier findings aneurysmal bone cysts, osteoid osteomas and osteoblastomas occur rarely. The prevalence of hemangioma correspond to the 27% reported in another MRI study among patients in Iran[6]. Much lower prevalence estimate of 10% in an early autopsy study likely reflects methodological issues and tells little about the real occurrence of these types of lesions[1]. The comparison with previous findings is complicated by either different methods to assess spinal lesions or by different target populations and sampling strategies. In other MRI studies on osseous lesions scans were commonly taken for a specific clinical reason within selected regions of the spine thus impeding plausible estimates of the prevalence of osseous lesions and their distribution in the spine[6, 7]. For example, observed differences in the sex distributions from previous findings regarding bone cysts[42, 43] may just be due to the very different samples underlying the findings. The prevalence of osseous lesions was lower in segments with high biomechanical demand. This strongly contrasts with the prevalence of degenerative diseases of the spine[40, 41]. This implicates different disease mechanisms which remain to be studied.

Our present results provide a new point of reference for the appraisal of the importance of benign osseous lesions and their subtypes. Entirely novel findings are

- Population-based prevalence estimates for different types of osseous lesions.
- For the first time we show the distribution across the segments of the entire spine, which was similar for the different types of osseous lesions.
- The age effects differ across gender.

Hormonal effects concerning estrogen and leptin could explain the strong rise for females in early years compared to the more linear increase for males. An explanation for the observed sex specific age effects remains speculative. First this finding should be replicated in other studies due to the potential instabilities of nonlinear effects before an attempt to find biological explanations showing more association between the single osseous lesions.

Furthermore, we were the first to systematically study the associations between osseous lesions and back as well as neck pain. Symptomatic osseous lesions are a very rare pathology that can present with persistent pain or neurological deficits[4, 12, 23, 24, 27, 28, 42, 44–49]. Osseous lesions have commonly not been regarded as a cause of pain and our findings support this assumption[28]. The association with back and neck pain was overall very weak, indicating a rather minor importance of osseous lesions with regards to the clinical back pain symptoms. A minor exception may be the modestly elevated relative risk related to the prediction of neck and spine pain in the past seven days with osseous lesions in the cervical spine and sclerosis. However, as we face a situation of extensive multiple testing (40 tests in total) the two statistically significant associations remain to be confirmed by other studies before drawing any substantial conclusions.

### Strength and limitations of the study

Strengths of our study are the large general population sample, which was assessed with whole-body MRI and a standardized interview to collect demographic and clinical data. Our sample is not affected by strong selection mechanisms commonly occurring in clinical settings. This increases the likelihood of our results generalizing to other Caucasian adult populations. Contrary to other available studies[1, 2, 6, 28, 50] a particular strength is that we did not focus on single types of osseous lesions but covered all major subtypes thus providing a broad picture of these types of lesions and their associations with pain. A limitation of this study is that we did not provide contrast-enhanced imaging or histological assessment of the detected osseous lesions. However, our analysis focused on osseous lesions, which can be adequately identified due to MRI. It should be noted that due to our population-based study setting we had not the ability to perform X-ray or comutertomography imaging for further characterization of osseous lesions.

Comparison of prevalence figures with and without statistical weights to account for selective non-response revealed negligible differences in our point estimates suggesting that known variables of relevance for MRI participation are not a relevant source of selection bias. The sample size is large enough to provide reliable estimates for frequent osseous lesions. However, in case of rare conditions such as osteoblastoma some ambiguity remains.

Limitations concern the cross-sectional design. We may not derive any directional conclusions on associations between osseous lesions and pain symptoms. Future studies should assess the stability of our findings in other populations, for example concerning potential age-sex interactions on the occurrence of osseous lesions. Furthermore, the association between osseous lesions and segmental diseases in organs within the innervation area of different spinal segments should be studied.

## Conclusion

Benign osseous lesions are common in the general population but of no clinical relevance for spinal pain. Most common types of benign osseous lesions are hemangiomas followed by lipomas. The prevalence of osseous lesions varied strongly across different regions of the spine, with peak levels between L1 and L3, followed by a second but lower peak between T4 and T8. and was also associated with age and gender. Our population-based data offer new insights and assist in judging the relevance of osseous lesions observed on MRIs of patients.
